# Multicentric investigation on the safety, feasibility and usability of the ABLE lower-limb robotic exoskeleton for individuals with spinal cord injury: a framework towards the standardisation of clinical evaluations

**DOI:** 10.1186/s12984-023-01165-0

**Published:** 2023-04-12

**Authors:** Mark Andrew Wright, Franziska Herzog, Anna Mas-Vinyals, Alfons Carnicero-Carmona, Joan Lobo-Prat, Cornelia Hensel, Steffen Franz, Norbert Weidner, Joan Vidal, Eloy Opisso, Rüdiger Rupp

**Affiliations:** 1 Institut Guttmann, University Institute attached to the Universitat Autònoma de Barcelona, Badalona, Barcelona Spain; 2 Universitat Autònoma de Barcelona, Bellaterra, Cerdanyola del Vallès, Spain; 3 Fundació Institut d’Investigació en Ciències de la Salut Germans Trias i Pujol, Badalona, Barcelona Spain; 4 Spinal Cord Injury Center, Heidelberg University Hospital, Schlierbacher Landstraße 200a, 69118 Heidelberg, Germany; 5 ABLE Human Motion S.L., Barcelona, Spain

**Keywords:** Spinal cord injury, Spinal cord disorder, Exoskeleton, Rehabilitation, Robotics, Safety, Feasibility, Usability, Gait, Study protocol, Standardisation

## Abstract

**Background:**

Robotic lower-limb exoskeletons have the potential to provide additional clinical benefits for persons with spinal cord injury (SCI). However, high variability between protocols does not allow the comparison of study results on safety and feasibility between different exoskeletons. We therefore incorporated key aspects from previous studies into our study protocol and accordingly conducted a multicentre study investigating the safety, feasibility and usability of the ABLE Exoskeleton in clinical settings.

**Methods:**

In this prospective pretest-posttest quasi-experimental study across two SCI centres in Germany and Spain, in- and outpatients with SCI were recruited into a 12-session training and assessment protocol, utilising the ABLE Exoskeleton. A follow-up visit after 4 weeks was included to assess after-training outcomes. Safety outcomes (device-related adverse events (AEs), number of drop-outs), feasibility and usability measures (level of assistance, donning/doffing-time) were recorded at every session together with changes in gait parameters and function. Patient-reported outcome measures including the rate of perceived exertion (RPE) and the psychosocial impact of the device were performed. Satisfaction with the device was evaluated in both participants and therapists.

**Results:**

All 24 participants (45 ± 12 years), with mainly subacute SCI (< 1 year after injury) from C5 to L3, (ASIA Impairment Scale A to D) completed the follow-up. In 242 training sessions, 8 device-related AEs (pain and skin lesions) were reported. Total time for don and doff was 6:50 ± 2:50 min. Improvements in level of assistance and gait parameters (time, steps, distance and speed, p < 0.05) were observed in all participants. Walking function and RPE improved in participants able to complete walking tests with (n = 9) and without (n = 6) the device at study start (p < 0.05). A positive psychosocial impact of the exoskeleton was reported and the satisfaction with the device was good, with best ratings in safety (participants), weight (therapists), durability and dimensions (both).

**Conclusions:**

Our study results prove the feasibility of safe gait training with the ABLE Exoskeleton in hospital settings for persons with SCI, with improved clinical outcomes after training. Our study protocol allowed for consistent comparison of the results with other exoskeleton trials and can serve as a future framework towards the standardisation of early clinical evaluations.

*Trial Registration* https://trialsearch.who.int/, DRKS00023503, retrospectively registered on November 18, 2020.

**Supplementary Information:**

The online version contains supplementary material available at 10.1186/s12984-023-01165-0.

## Background

Spinal cord injury (SCI) is a life-changing condition that affects multiple body systems resulting in sensory and motor impairments very often associated with long-term immobility [1–3]. Achieving independent ambulation has a high relevance for participation in social and professional life [1, 4]. In the past 10 years, one of the most notable technological developments to support this has been the creation of lower-limb robotic exoskeletons. These devices aim to allow individuals with SCI to perform motor learning-based training with multiple repetitions of the locomotor task and minimal physical burden to therapists, with a view to allowing independent continuation of the training in a community setting in the future [2, 5]. A number of exoskeletons are now certified for use in a clinical setting which previously underwent an initial study as part of the certification process to validate their safety and feasibility in the intended population [2, 6, 7].

Despite the widely recognised need for safety and feasibility testing, there is significant variability in the study protocols used within these studies [8]. While most studies include participants 6 months or more after SCI, the participants' level and severity of injury are often highly variable, with vastly different inclusion and exclusion criteria reported even with the same device [9]. Concern has previously been expressed about the marked variability in the different methods and levels of detail used to report safety aspects, with events such as fractures not consistently considered as serious adverse events in previous trials [9, 10]. Accordingly, future study protocols should facilitate more meaningful comparisons between studies and should be better structured to identify risk concerns such as falls, fractures, and long-term adverse effects of using exoskeletons [8, 9]. It has also been highlighted that future studies should broaden their scope to include reporting on changes in areas related to quality of life (QoL) [8].

The aim of our multicentre investigation was to evaluate the ABLE Exoskeleton regarding its safe and feasible use in clinical settings. Special emphasis was placed on the development of a transferable study protocol that takes the key aspects identified in previous literature into account and therefore allows for the comparison of study results.

## Methods

### Study objectives

The primary objective of this study was to determine the safety, feasibility and usability of the ABLE Exoskeleton for individuals with traumatic and non-traumatic SCI in a hospital setting during a 4–6-week training programme. The secondary objectives were to assess the effect of the ABLE Exoskeleton on gait and functional outcomes, the rate of perceived exertion, psychosocial impact and user satisfaction.

### Study design

We conducted a prospective pretest–posttest quasi-experimental study in two European SCI centres (Institut Guttmann, Badalona, Spain and Heidelberg University Hospital, Heidelberg, Germany) from November 2020 until October 2021. The individual pre–post training period covered 4–6 weeks. The clinical trial was approved by the responsible local ethics committees and competent authorities of Spain and Germany, respectively (EUDAMED No.: CIV-20-07-034264). The study was conducted in accordance with the requirements of ISO 14155:2011 and European Regulation MDR 2017/745 on medical devices.

### ABLE Exoskeleton

**Fig. 1:**
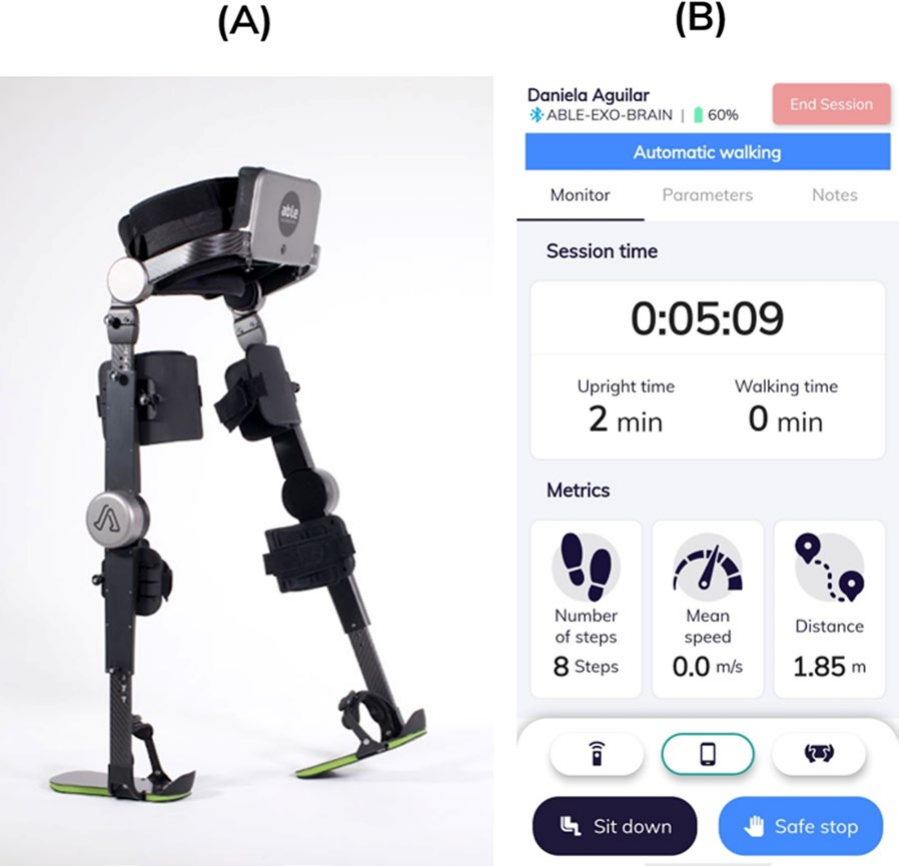
Overview of the hard- and software of the ABLE Exoskeleton. **A** The ABLE Exoskeleton with the knee motors and passive hip joints. **B** The graphical user interface of the ABLE Care mobile app

The ABLE Exoskeleton (Fig. 1) is a wearable lower-limb robotic exoskeleton weighing 9.8 kg and consisting of a rigid brace that attaches to the torso, legs and feet of the user via straps and supports. The size of the exoskeleton segments can be adjusted to fit users with a height of 150–190 cm and a maximum weight of 100 kg. This bilateral exoskeleton has two battery-powered motors that assist flexion–extension movements of each knee joint, and two non-motorized, passive hip joints that allow free movement in the sagittal plane. With its knee motors, it actively assists a person to stand up, walk and sit down. Steps with the associated knee flexion–extension movements during swing phase are triggered either manually by the therapist or automatically by the user. The manual step trigger is activated by means of pressing either of the pushbuttons (left for triggering the left knee flexion–extension movement and right for the right one) that are located on the lumbar segment of the exoskeleton. An exoskeleton user is encouraged to maintain an upright posture during gait and to utilise momentum inducing weight shifts synchronously to the triggering of the stepping action on the respective side. In the automatic step initiation mode, the exoskeleton autonomously seeks to determine the time instant when the user wants to take a step, detected as a change in the thigh angular velocity measured with an inertial measurement unit on the leg brace of the exoskeleton. When this signal change is detected, a step is triggered on the leg that is behind. Due to the passive hip joints of the exoskeleton, in people with no or very limited voluntary hip movements, hip flexion is generated by forward-rotational movement of the pelvis. An analysis of the gait pattern used whilst walking with the ABLE exoskeleton has been analysed in a previous study [11]. Note that the aim of having the different walking modes is to adapt the exoskeleton to the users' needs. Once participants are familiar with the device and have learned how to move to initiate steps, it is possible to change the step initiation mode from manual to automatic walking. The device is to be used with a walking aid (cane, crutch or walker) for supporting a stable body position of the users who typically have restrictions to fully stabilize their trunk and always under the supervision of a trained therapist. The device comes with a smartphone with the pre-installed software application ABLE Care (Fig. 1) that allows the therapist to configure and monitor the exoskeleton during the therapy session.

### Study protocol & framework

To address the need for better conformity of exoskeleton trials we based our study protocol on a systematic literature search of studies investigating the safety and/or feasibility and/or usability of robotic lower-limb exoskeletons. A total of 13 studies were analysed to identify the most common in- and exclusion criteria, training methodology and outcome measures (for an overview see Additional file 1).

#### In- and exclusion criteria

The inclusion and exclusion criteria of the study (see Table 1) were largely identified by using criteria corresponding to other exoskeleton safety and feasibility trials. Most studies use the American Spinal Injury Association Impairment Scale (AIS) of the International Standards for Neurological Classification of SCI (ISNCSCI) [12] for inclusion, however, in order to not limit the recruitment process we decided to add the criterion “sufficient arm strength to support body weight on a walking frame” as in previous studies [4, 13, 14]. Screening evaluations for fracture risks in previous studies ranged from Dual Energy X-Ray Absorptiometry (DXA) bone density scans [15, 16] to simply a medical history of osteoporosis [13, 14, 17]. Considering the variability in measures used, we decided to exclude any individual with a history of lower-limb fragility fractures in the last 2 years [4], and/or, who had 5 or more risk factors present for fragility fractures as cited by Craven et al. [18]. It was considered that these criteria were extensive enough to cover risks, whilst also being accessible to all SCI centres without immediate access to DXA scans.

**Table 1:** Inclusion and exclusion criteria.

Inclusion Criteria	Exclusion Criteria
∙ 18–70 years of age ∙ Traumatic and non-traumatic SCI ∙ AIS grade A to–D with sufficient arm strength to support body weight on a walking frame ∙ Currently receiving treatment as an inpatient or outpatient at one of the investigational sites ∙ Ability to give informed consent	∙ WISCI II [19] without exoskeleton of > 16 ∙ History of lower-limb fragility fractures in the last 2 years ∙ 5 or more risk factors present for fragility fractures as stated by Craven et al. [18] ∙ Deterioration > 3 points of the total ISNCSCI motor score within the last 4 weeks ∙ Spinal instability ∙ Modified Ashworth scale [20] > 3 in lower limbs ∙ Unable to tolerate 30 min standing without clinical symptoms of orthostatic hypotension ∙ Unable to perform a sit-to-stand transfer or stand in the device with assistance ∙ Psychological or cognitive issues that do not allow the participant to follow the study procedures ∙ Known pregnancy or breastfeeding ∙ Any neurological condition other than SCI ∙ Medically unstable (Unstable CVS, hemodynamic instability, untreated hypertension (SBP > 140, DBP > 90 mmHg), unresolved DVT, uncontrolled AD) ∙ Severe comorbidities (any condition that a physician considers to not be appropriate to complete participation in the study) ∙ Ongoing skin issues (Grade I or higher on the European Pressure Ulcer Advisory Panel (EPUAP) scale [23] on areas that will be in contact with the exoskeleton) ∙ Height, width, weight or other anatomical constraints (such as leg length differences) incompatible with the device ∙ Insufficient ROM for device preventing a participant to achieve a normal gait pattern or to complete a sit-to-stand/stand-to-sit transition (hip joint: insufficient ROM for step length, knee joint: insufficient ROM for standing, ankle: insufficient ROM to reach neutral position)

Abbreviations are defined in the Abbreviations section

#### Training methodology

Sampling was completed by the pre-screening of all in- and outpatients. All patients who met the inclusion criteria and did not present with criteria for exclusion were asked to participate in the clinical trial. After screening for in- and exclusion criteria, a baseline visit was completed, followed by the training programme and a post-training visit. Four weeks after the final training session a follow-up visit was performed by phone interview due to the COVID-19 restrictions.

**Fig. 2:**
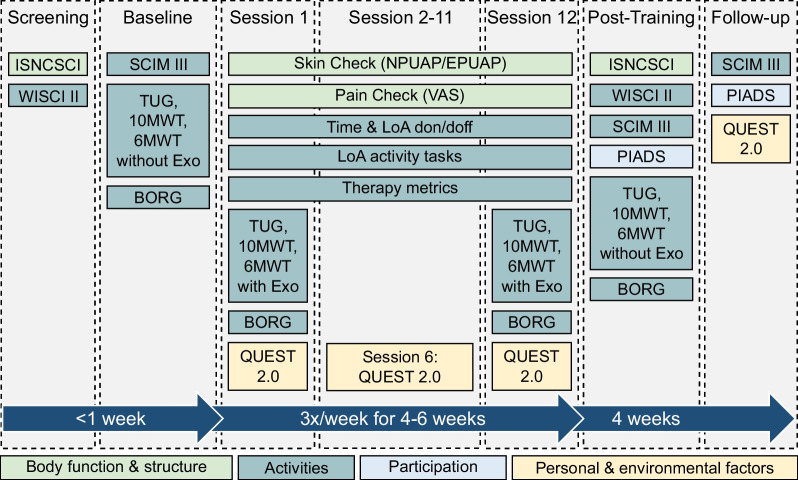
Overview of all study visits and measurements based on the International Classification of Functioning, Disability and Health (ICF) domains. Abbreviations are defined in the Abbreviation section

The training programme with the ABLE Exoskeleton (Fig. 2) consisted of three sessions a week over 4 weeks to achieve a total of 12 sessions. Missed sessions could be made up within 6 weeks after baseline. The training sessions were scheduled for 60 min to include adjustments, donning and doffing time and data collection time. Therapy time (time spent standing, walking or sitting in the exoskeleton) was intended to be at least 30 min. Each session was carried out by at least one trained therapist plus an additional therapist or assistant if required. During the first training sessions, participants were educated on the operating mechanisms of the exoskeleton and guided through the basic use of the device: sit-to-stand, standing, weight shifting, stand-to-sit and walking, using crutches or frame as deemed appropriate for each individual. The step initiation mode was individually chosen according to the participant’s ability and may have changed as the study progressed. If the therapist considered it to be safe, the participant was required to complete the following activity tasks during every session with as little assistance as possible from the therapist: sit-to-stand, walk 10 metre, turn 180° and stand-to-sit. These predefined activity tasks were implemented to achieve a better comparability of the feasibility in different individual users of the exoskeleton.

#### Outcome measures

The primary endpoints for safety were the number of serious adverse events (SAEs), adverse events (AEs) and study drop-outs related to the use of the device. SAEs and AEs were classified systematically according to ISO 14155:2011, MEDDEV 2.7/3 and MDCG 2020-10/1. AEs were defined in four categories: (1) device-related (AEs that have occurred as a direct result of the device itself), (2) procedure-related (AEs that occurred as a result of the activities performed in training, but were not caused by the device), (3) disease-related (AEs that occurred as a result of the underlying health conditions), and (4) other causes or undetermined relation. In addition to these definitions, we considered device deficiencies as AEs without a medical occurrence. Within these categories the degree of relation was rated by the investigators as ‘related’, ‘probably related’, ‘possibly related’, ‘unlikely related’ and ‘not related’. Only those AEs rated as ‘related’ were defined as device/procedure/disease-related AEs.

Specific AE monitoring that was also noted in previous studies involving exoskeleton devices included checks for skin lesions, assessment of pain levels using a visual analogue scale (VAS) pre- and post-each session [1, 2, 4, 6, 13, 15, 16, 21, 22], frequency of falls and any event that required medical intervention [6, 21, 22]. The following assessments were performed before and after each training session in this study: screening of medical records for AEs; skin checks for signs of skin lesions on parts of the body in contact with the exoskeleton graded as I or higher with the European Pressure Ulcer Advisory Panel (EPUAP) scale [23]; and, recording of the presence, location and severity (VAS) of pain. Any falls during training sessions were also documented. Furthermore, any requirement to have a medical review after the last session and its relation to the device was registered.

For feasibility and usability, the ease of donning and doffing the device was measured by the time and the level of assistance (LoA) the participants needed to conduct these activities, with the aim to conduct donning and doffing as independently and as time-efficiently as possible. Several studies have previously charted LoA, however defining this can be a very subjective process, with variabilities between grading [6, 13, 15, 16, 21, 24]. As a result, we combined the best practices identified in previous studies to produce a LoA scale (see Table 2). The LoA was rated by the therapists in the following stages: total, maximum, moderate and minimal assistance, supervision and independence. These stages were also used to assess the LoA to perform the predefined activity tasks. The study participants’ ability to use the device was also assessed by the therapy metrics recorded by the device: number of steps, standing and walking time, speed and distance.

**Table 2:** Level of Assistance definitions

Level of Assistance	Don/Doff	Therapy activity task
Total assistance	Participant performs 0–25% of the effort to don/doff the exoskeleton Participant is essentially reliant on the trainer to perform all aspects of the donning/doffing	Participant performs 0–25% of the effort to use the exoskeleton Two therapists are required to support the participant in the device at all times
Maximum assistance	Participant performs 25–50% of the effort to don/doff the device Participant needs maximum assistance to transfer to device and position legs but may be able to adjust the thigh straps	Participant performs 25–50% of the effort to use the device Participant needs maximum assistance from the therapist to remain balanced
Moderate assistance	Participant performs 50–75% of the effort to don/doff the device Participant needs moderate assistance to transfer to device and position legs but may be able to adjust the thigh and shin straps	Participant performs 50–75% of the effort to use the device The therapist has both hands on the participant or device at all times to provide occasional guidance or balance support
Minimal assistance	Participant performs > 75% of the effort to don/doff the device Participant can transfer to device and adjust straps but may need help to position legs	Participant performs > 75% or more of the effort to use the device The therapist has one hand on the participant or device for infrequent guidance or balance support
Supervision	The therapist is not touching the participant but may provide verbal prompts or contact guarding to ensure safety	The therapist is not touching the participant but is close enough to provide support for balance or guidance as needed
Independent	Participant is fully independent donning/doffing device	The participant is fully independent while using the device and the therapist does not provide any assistance

Level of Assistance was defined separately for Donning/Doffing of the device and for the therapy activity tasks

Secondary endpoints aimed to identify the effect of the exoskeleton training on participants’ gait and function, rate of perceived exertion (RPE), user satisfaction and psychosocial impact. Gait was assessed by conducting the following standardised walking assessments: Walking Index for Spinal Cord Injury (WISCI) II, Timed Up and Go (TUG), 10 Metre Walk Test (10MWT) and 6 Minute Walk Test (6MWT) as performed in numerous previous exoskeleton trials [1, 2, 4, 14–17, 21, 22, 24, 25]. If deemed safe by the therapist, walking assessments were completed without the device at baseline and post-training, and with the device during sessions 1 and 12. Standardised rest breaks were included in these sessions to ensure results were reliable and valid. If any training sessions between 2 and 11 were missed, the participant was still given the opportunity to complete walking tests at session 12 with the exoskeleton before the end of the 6-week training programme. For the 6MWT, a course of 50 m was marked out, with participants turning 180° once arriving at 50 m. As in previous studies, the RPE was measured with the BORG-scale after the 6MWT [14]. Function in respect to independence was evaluated by the Spinal Cord Independence Measure (SCIM) III as in previous studies [22]. Most of the assessments used in prior exoskeleton trials covered the function and activity domain of the International Classification of Functioning, Disability and Health (ICF) (see Fig. 2). To provide a meaningful insight of exoskeleton use for all domains of the ICF we placed a special emphasis on the inclusion of Patient-Reported Outcome Measures (PROMs). Two additional questionnaires were therefore included, the Quebec User Evaluation of Satisfaction with assistive Technology (QUEST 2.0) and the Psychosocial Impact of Assistive Devices Scale (PIADS). The QUEST 2.0 measures the satisfaction with an assistive device and has been used in previous studies with exoskeletons [13, 26, 27]. The PIADS assesses the psychosocial impact of a device on a sum scale from -78 (negative impact) to + 78 (positive impact) [28]. To the best of our knowledge, the PIADS has not been implemented in previous studies with mobile exoskeletons used in a clinical setting by people with SCI [29–31]. Therapists’ satisfaction was measured once with the QUEST 2.0 at the end of the study period.

### Statistical analysis

Quantitative variables were summarised using standard descriptive statistics (median, interquartile range (IQR), mean, standard deviation (SD), minimum and maximum). Qualitative variables including (S)AEs were described using group sizes and frequencies. The differences between pre–post training outcome measures were analysed using the non-parametric Wilcoxon-test. For multiple measures, the Friedman test and Wilcoxon post-hoc test with adjusted Bonferroni–Holm correction were calculated with IBM SPSS Statistics 28.0. P-values p ≤ 0.05 were considered statistically significant.

## Results

Twenty-four individuals with SCI with neurological levels of injury ranging from C5 to L3 participated in the study (see Table 3). For complete injuries (AIS A) only thoracic lesions from T5 to T12 were present in our study population. Motor complete injuries (AIS grades A and B) were present in 16 participants and 8 were classified as motor incomplete (AIS grades C and D). Twenty individuals sustained the injury within the last year (acute or subacute), while 4 individuals were in the chronic stage (onset of paralysis injury more than 1 year). On average, participants were 45 ± 12 years old and mostly male (70.8%). 58.3% of participants had a traumatic SCI while 41.7% had a non-traumatic SCI.

**Table 3:** A detailed overview of the characteristics of each study participant

Participant	Sex	Age range	Height ranges	Weight ranges	Days from injury at day of IC	Injury aetiology	NLI	AIS	LEMS screening	LEMS post-training	WISCI screening	WISCI post-training	SCIM mobility baseline	SCIM mobility follow-up
1	Male	61–70	176–180	96–100	22	NT	L3	B	20	21	1	6	17	19
2	Female	61–70	161–165	66–70	15	NT	T10	D	39	44	0	8	15	19
3	Female	61–70	166–170	56–60	36	NT	T11	A	0	0	0	0	16	17
4	Female	18–30	166–170	46–50	2184	T	T7	A	0	0	6	5	19	18
5	Female	31–45	171–175	56–60	670	NT	T12	B	7	7	13	13	21	21
6	Male	46–60	176–180	86–90	2941	T	T6	A	0	0	0	0	14	18
7	Female	31–45	161–165	86–90	6202	T	T9	A	0	0	0	0	14	13
8	Male	31–45	176–180	71–75	115	T	T12	A	0	0	6	6	15	17
9	Male	46–60	176–180	66–70	168	T	T10	A	0	0	0	3	15	17
10	Male	31–45	181–185	71–75	54	T	T10	A	0	0	1	4	17	19
11	Male	31–45	171–175	51–55	55	NT	T1	D	46	–	8	–	18	22
12	Male	46–60	176–180	76–80	104	NT	T7	D	46	–	4	–	18	20
13	Male	31–45	186–190	71–75	92	T	L3	B	21	21	9	9	18	23
14	Male	31–45	161–165	51–55	120	T	C5	B	0	0	3	3	13	12
15	Male	31–45	176–180	81–85	123	T	T5	A	0	0	1	3	14	15
16	Female	31–45	161–165	56–60	64	T	T11	D	24	27	5	9	18	21
17	Male	31–45	176–180	71–75	71	T	L3	B	18	28	9	12	18	24
18	Male	46–60	181–185	71–75	179	NT	T5	D	38	38	13	13	21	21
19	Male	31–45	176–180	76–80	79	NT	T7	D	40	46	13	16	16	31
20	Male	31–45	181–185	76–80	55	T	T11	A	0	0	3	3	18	19
21	Male	46–60	181–185	71–75	107	T	T6	C	19	22	3	6	16	19
22	Male	31–45	181–185	71–75	94	T	T7	B	0	0	3	5	18	19
23	Male	31–45	186–190	76–80	111	NT	L1	D	36	40	4	8	17	21
24	Female	31–45	161–165	76–80	185	NT	T11	B	0	1	1	1	15	16

Ranges of age, height and weight are given to ensure anonymity of the study participants. The neurological level and the severity of the lesion are given together with the lower extremity motor scores, the dependency on walking aids, and the functional status before and after the training

*IC* informed consent; *NLI* neurological level of injury; *AIS* American Spinal Injury Association (ASIA) Impairment Scale; *T* traumatic; *NT* non-traumatic; *LEMS* Lower Extremity Motor Score according to the International Standards for Neurological Classification of SCI (ISNCSCI) [12]; *WISCI* Walking Index for Spinal Cord Injury, measured without the ABLE Exoskeleton; *SCIM* Spinal Cord Independence Measure

**Fig. 3:**
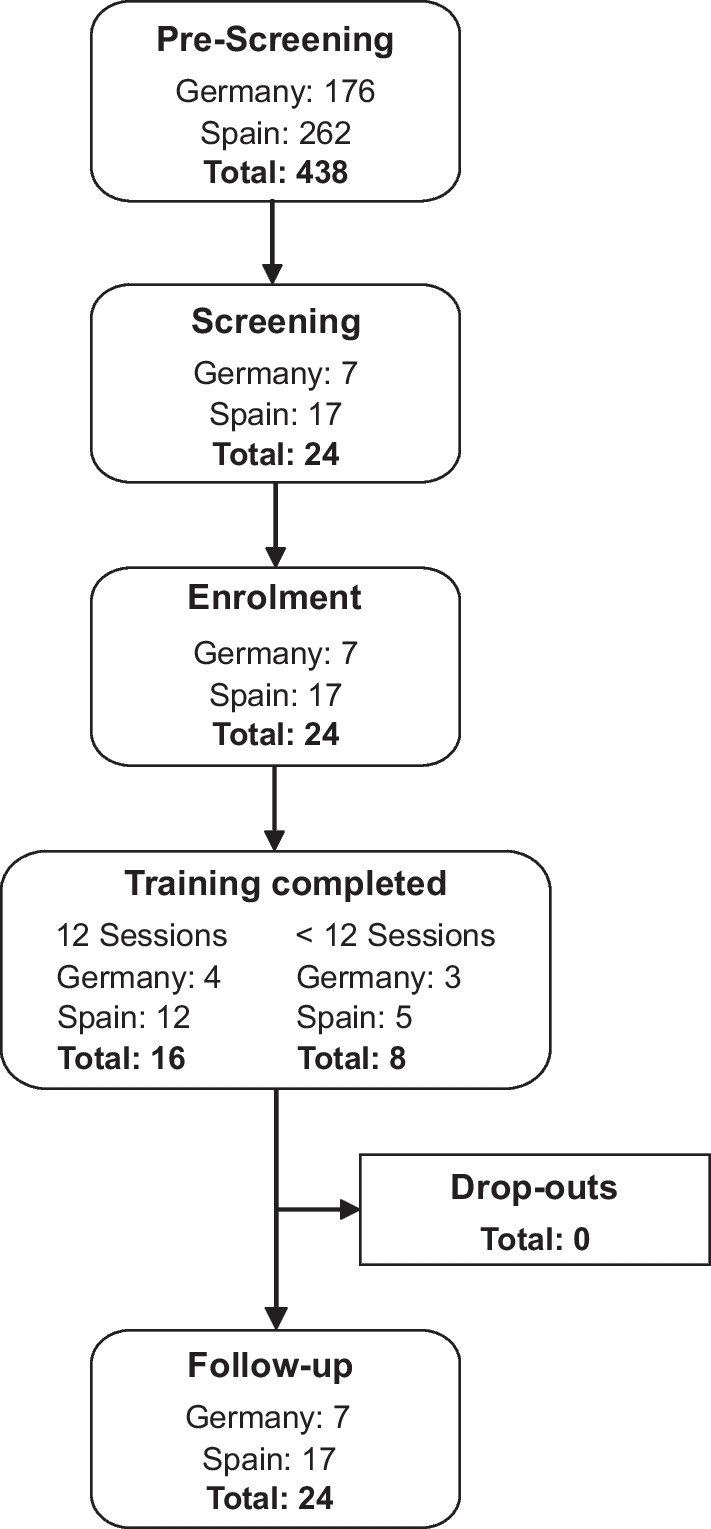
Study flow chart of recruited participants in line with the STROBE statement (http://www.strobestatement.org)

On average 10.1 ± 3.5 training sessions were completed. Sixteen participants (66.7%) completed all 12 training sessions, 2 participants missed one session (8%), 2 participants missed three and five sessions respectively (8%), and 4 participants completed less than six training sessions (16.6%). Nearly all missed sessions were due to health reasons unrelated to training, along with required maintenance periods for the device. One participant could not complete session 12 due to training-related back pain, which recovered after 1 week without the need for medical treatment. All participants remained in the study until the follow-up visit, thus there were no drop-outs (see Fig. 3).

### Primary study outcome

#### Safety outcome measures

A total of 81 AEs were reported during the 242 sessions performed throughout the study across the two sites. Eight (9.9%) of these events, occurring in 6 participants, were classified as device-related AEs, i.e. rated as ‘related’ to the device by the assessors. None of the AEs were rated as serious. Other reported events without a medical occurrence were classified as device deficiencies according to ISO 14155:2011. No falls or fractures occurred.

Table 4 summarises the types of AEs that occurred during the study, as well as the percentage of participants affected. The most reported AEs corresponded to skin lesions (33.3%) and pain (27.2%) in 13 and 10 participants, respectively. Three participants experienced isolated episodes of orthostatic hypotension either at the start of a session before standing up, or upon standing up, with one of the participants unable to continue with the remainder of that session. Medication was changed for two of these participants prior to the subsequent session, whilst the third participant remained on the same medication management. All three participants were able to continue with the training sessions as planned without further problems.

**Table 4:** Overview of adverse events (AEs)

Type of AE	Number (% out of total AEs)	Device-related (% out of AE-type)	Procedure-related (% out of AE-type)	Underlying disease-related (% out of AE-type)	Other causes or undetermined relation (% out of AE-type)
Skin lesion	27 (33.3)	5 (18.5)	0 (0)	1 (3.7)	21 (77.8)
Pain	22 (27.2)	3 (13.6)	6 (27.3)	4 (18.2)	9 (40.9)
Neuropathic pain	9 (11.1)	0 (0)	0 (0)	9 (100)	0 (0)
Urinary tract/gastrointestinal infection	8 (9.9)	0 (0)	0 (0)	5 (62.5)	3 (37.5)
Fatigue	3 (3.7)	0 (0)	3 (100)	0 (0)	0 (0)
Spasticity	3 (3.7)	0 (0)	0 (0)	3 (100)	0 (0)
Hypotension	5 (6.2)	0 (0)	3 (60.0)	2 (40.0)	0 (0)
Inflammation	2 (2.5)	0 (0)	0 (0)	0 (0)	2 (100)
Other	2 (2.5)	0 (0)	1 (50)	0 (0)	1 (50)
Total	81 (100)	8 (9.9)	13 (16.0)	24 (29.6)	36 (44.4)

Type and number of AEs that occurred during the clinical investigation

Most of the device-related skin lesions were mild pressure injuries (grade I according to the EPUAP scale) (3/5) and were caused by the contact between the participants’ body and the exoskeleton structure. These were located at the lower back at the level of the already padded lumbar module and at the instep area under the foot straps. All skin injuries were resolved without further complications (average recovery time of 5 days) by re-adjusting the fitting of the exoskeleton segments and, when needed, adding extra padding to the critical skin areas. Two device-related skin lesions occurring in the same participant were rated as moderate (grade II sacral pressure injury). These led to a training pause of 8 weeks. Due to an insufficient effect of adding extra padding, the training was resumed by using a hydrocolloid and a foam plaster over the critical skin areas during training. Although the skin of this study participant was intact at screening, it is important to note that this participant had a known history of pressure injuries at the right ischial tuberosity before involvement in the clinical study and therefore had a generally high risk for developing pressure injuries. Of the 21 skin issues that were assigned to other causes, 11 were rated as probably device-related. Most of these cases (10/11) were non-blanchable redness (EPUAP grade I) that appeared directly after the exoskeleton training and recovered the same day without the need for any medical treatment. Mild bruising on the shin was noted before the 11th session for one participant, however, this was resolved by the end of training. It was not clear if this was device-related given that he was involved in many other therapy activities, and that he did not present with this bruising in any of the 10 previous sessions. Two skin lesions on the lower leg were rated as “possibly and unlikely” device-related, two skin lesions on the foot were classified as “probably or possibly related” to the medical procedure and one redness on the back could have a “possible relation” to the disease condition. Five skin lesions happened between the training sessions and were not related to any of the three categories (device-, procedure- or disease-related).

Most of the device-related pain issues were mild (2/3) and were resolved in the same or subsequent session. One event of a device deficiency caused a pain score of VAS 8/10. The exoskeleton lost connection to the mobile application and as a consequence, the participant needed to be assisted to sit down on the chair with extended legs which led to pain in the posterior part of the knee. The pain resolved immediately after the exoskeleton was doffed and the participant sustained no further issues.

Including the above-mentioned device deficiency that led to a device-related AE, a total of 16 device deficiencies have been reported during the clinical investigation, of which, 15 were classified as malfunctions and one as a user error of the participant. Two of these malfunctions corresponded to mechanical issues of the exoskeleton, while the rest were related to the exoskeleton firmware, the mobile app software or a combination of both. All device deficiencies were promptly addressed and resolved by the manufacturer and the changes made to the devices were reported to the regulatory agencies.

#### Feasibility and usability outcome measures

Donning and doffing of the device required an average total time of 6 min and 50 s (Table 5).

**Table 5:** Time to Don and Doff the ABLE Exoskeleton

	Mean ± SD	Median; IQR	Range
t_DON_ (min:sec)	04:43 ± 1:52	04:15; 2:18	2:00–12:30
t_DOFF_ (min:sec)	02:07 ± 1:10	01:43; 01:32	0:30–7:24
t_DON_ + t_DOFF_ (min:sec)	06:50 ± 2:50	06:05; 3:39	2:30–15:21

Mean, median and range of time to Don and Doff the device are shown

**Fig. 4:**
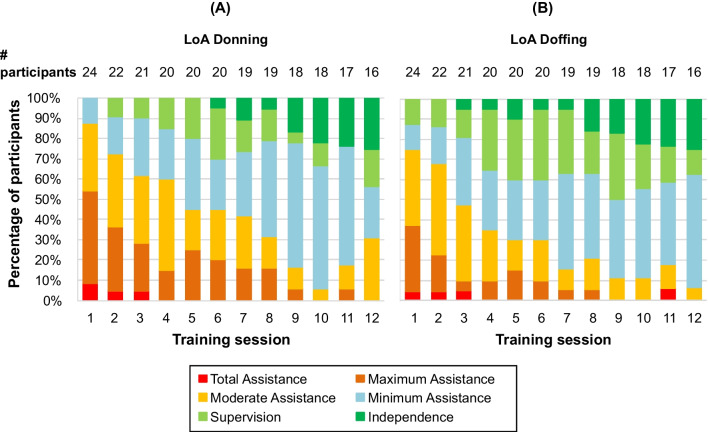
Progression of the Level of Assistance in donning and doffing. Level of Assistance (LoA) for **A** Donning and **B** Doffing is shown over the course of the training sessions

There was a significantly reduced LoA over the training sessions for donning and doffing (p < 0.001 for 16 participants who completed all 12 training sessions). At the end of the training, the majority of participants (68.8%) were able to complete both donning and doffing either independently (25%), with supervision only (18.8%) or with minimal assistance (25%) (see Fig. 4).

**Fig. 5:**
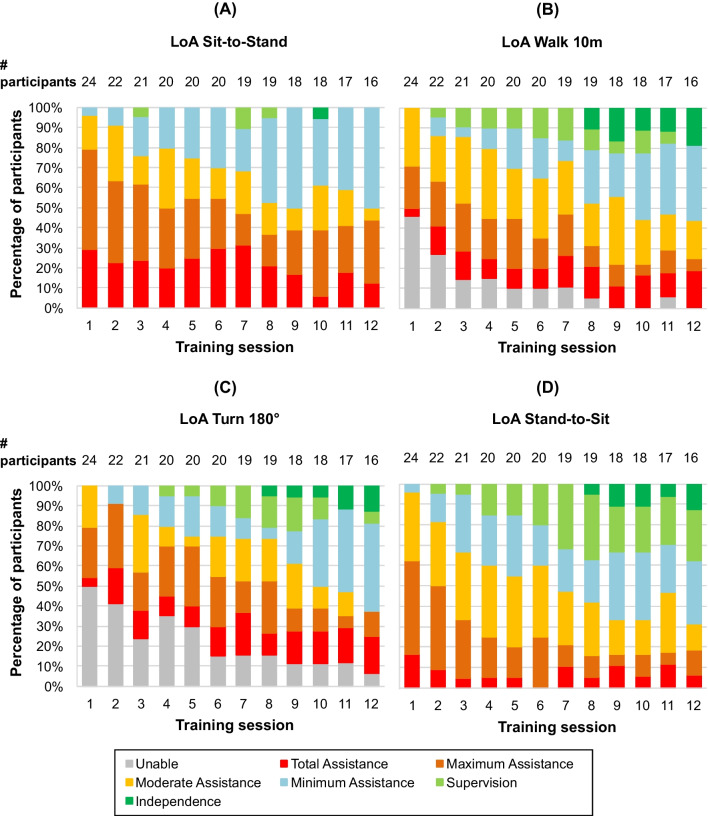
Progression of the Level of Assistance in therapy tasks. The Level of Assistance (LoA) for **A** Sit-to-Stand, **B** Walk 10m, **C** Turn 180°, and **D** Stand-to-Sit tasks is shown over the course of the training sessions

Independence to carry out the activity tasks (see Fig. 5) in the device (Sit-to-stand, Walk 10m, Turn 180º, Stand-to-sit) increased significantly for all participants as the training progressed (p < 0.001 for 16 participants, who completed all 12 training sessions). In session 1, 54.2% of the 24 participants were able to walk 10 m with the device, and required total assistance (4.2%), maximum assistance (20.8%) or moderate assistance (29.2%). By session 12, the 16 participants who completed all training sessions were able to walk 10 metre with the device, and 56.3% of them required low levels of assistance (37.5% with minimum assistance and 18.8% with total independence).

In session 1 all participants were able to complete sit-to-stand and stand-to-sit tasks, with one participant able to do so with a minimum level of assistance. By the end of the training programme, 50.0% of the 16 participants who completed session 12 were able to Sit-to-Stand with minimum assistance, and 68.8% performed stand-to-sit with low levels of assistance (31.3% minimum assistance 25.0% supervision, 12.5% total independence). The independence to carry out the 180º turn task also improved throughout the training programme, with 62.5% of the participants completing this task with minimum assistance (43.8%), supervision (6.3%) or total independence (12.5%) in session 12.

### Therapy metrics

**Fig. 6:**
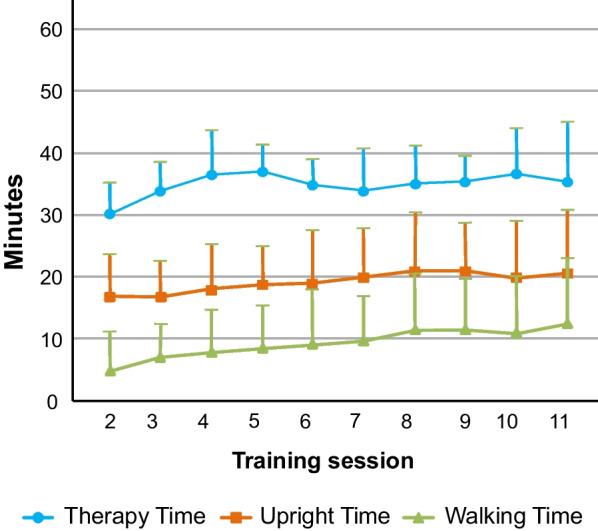
Average therapy time progression. The average total therapy time (time spent standing, walking or sitting in the exoskeleton) together with the standing and walking time for each session of the training programme of the 17 participants that completed sessions 2–11 is shown. Error bars represent standard deviation

A mean therapy time per session of 34.8 min ± 6.5 (median 34.9, IQR 6.0 min, range 13.1–57.5 min) was achieved. Figure 6 shows a clear trend towards higher standing and walking times throughout the training programme. As stated previously, sessions 1 and 12 were used to perform outcome measures with the exoskeleton, including standardised rest breaks, therefore therapy metrics for these sessions were not considered. Participants’ mean walking time in session 11 was 2.6 times higher compared to session 2 for those who completed these training sessions (n = 17). Besides the 16 participants who completed all 12 sessions, one additional participant completed sessions 2–11 (only missing session 12 due to back pain) and was therefore considered for this calculation of therapy metrics. The mean number of steps, speed and distance all increased by 3.0, 1.8 and 2.9 times respectively, from session 2 to 11.

**Fig. 7:**
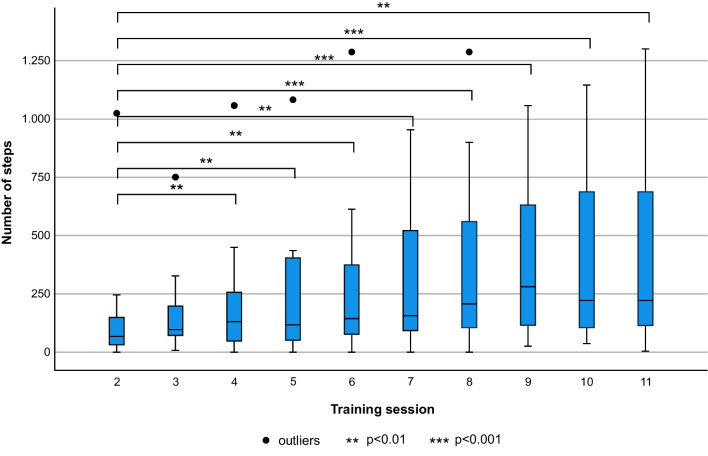
Progression of number of steps with the ABLE Exoskeleton. A boxplot of the number of steps for the 17 participants, who completed sessions 2–11, is shown

All participants who completed sessions 2–11 showed a significant improvement in walking time, number of steps, speed and distance from beginning to the end of the training programme (p < 0.001) (Table 6, Fig. 7). No significant differences were found between successive sessions.

**Table 6:** Overview of therapy metrics

	Session	Median (IQR)	Mean (SD)	Range	p-value
Walking time (min)	2	3.0 (5.0)	4.8 (6.5)	0–27	< 0.001
	6	7.0 (13.0)	9.1 (8.9)	0–31	
	11	8.0 (17.0)	12.4 (10.7)	0–32	
Steps (n)	2	68.0 (128.0)	138.8 (239.7)	0–1025	< 0.001
	6	144.0 (335.0)	282.1 (322.8)	0–1288	
	11	222.0 (610.0)	422.8 (415.5)	4–1301	
Speed (m/s)	2	0.1 (0.1)	0.1 (0.1)	0–0.3	< 0.001
	6	0.1 (0.1)	0.1 (0.1)	0–0.4	
	11	0.2 (0.1)	0.2 (0.1)	0.1–0.3	
Distance (m)	2	23.0 (59.7)	55.2 (111.9)	0–476.5	< 0.001
	6	41.0 (145.1)	108.8 (150.4)	0–602.5	
	11	87.1 (265.3)	157.8 (164.1)	1.4–491.0	

Median, interquartile range (IQR), mean, standard deviation (SD), range and Friedman-test p-value of walking time, steps, speed and distance of session 2, 6 and 11 for 17 participants, who completed sessions 2–11

### Secondary outcome measures

#### Activity outcomes

An increase was seen in the number of participants who were able to complete all walking tests with the device in session 12 compared to session 1 (Table 7). In addition to the 16 participants who completed all 12 sessions, 2 participants missed 2 and 6 sessions respectively, but still completed walking tests with the exoskeleton at sessions 1 and 12 and were therefore included in final calculations. Participants who were able to complete the TUG, 10MWT and 6MWT at sessions 1 and 12 showed a significant improvement (p < 0.01), with average gait speed during the 10MWT being 2.1 times faster in session 12 (0.3 [m/s] ± 0.1, 0.1–0.5) compared to session 1 (mean 0.1 [m/s] ± 0.1, 0–0.3). These participants also walked a greater distance (an average of 1.9 times further) during the 6MWT at the end of the training (Table 7).

**Table 7:** Walking test results with the ABLE Exoskeleton at session 1 and 12

	Session	Number of participants completed (%)	Number of participants completed session 1 and 12 (%)	Median (IQR)*	Mean (SD)*	Range*	p-value*
TUG (s)	S. 1	12 (50)	9 (37.5)	147.0 (133.0)	167.0 (89.2)	70–352	0.004
	S. 12	17 (70.8)		86.0 (45.0)	78.2 (27.7)	44–128	
10MWT (s)	S. 1	13 (54.2)	10 (41.7)	74.5 (115.0)	120.2 (102.7)	32–343	0.002
	S. 12	17 (70.8)		35.0 (30.0)	41.3 (20.4)	22–81	
6 MWT (m)	S. 1	10 (41.7)	9 (37.5)	33.0 (55.0)	48.4 (35.8)	10–118	0.004
	S. 12	15 (62.5)		100.0 (109.0)	94.1 (37.0)	22–131	
BORG (6–20)	S. 1	14 (58.3)	11 (45.8)	15.0 (7.0)	14.3 (4.2)	6–19	0.016
	S. 12	18 (75)		11.0 (5.0)	11.9 (3.3)	6–17	

Results of the Timed-up and Go test (TUG), the 10 Metre Walk Test (10MWT), the 6 Min. Walk Test (6MWT) and the BORG-scale at sessions 1 and 12 are shown

*Only for participants who completed both session 1 and 12

The BORG-scale was measured after the 6MWT even when the test was aborted (Table 7). A significant reduction of the RPE was seen for individuals who performed this test in session 1 and session 12 with the exoskeleton (p < 0.05). One participant had to abort all 3 walking tests at session 12, but still completed the BORG-scale.

This trend was also observed in the walking tests completed without the device. There were 3 more participants who were able to complete the walking tests without the device at post-training compared to baseline, with significant improvements in TUG and 6MWT (p < 0.05). The BORG-scale was measured without the exoskeleton for 12 (50%) participants post-training compared to 8 (33%) at baseline, with no change seen in scoring. The median WISCI II score changed significantly from 3.0; IQR 6.0 (mean 4.3 ± 4.5; range 0–13) pre-training to 5.5; IQR 6.0 (mean 6.1 ± 4.6; range 0–16) post-training (p < 0.01). Participants showed significant positive changes in the total SCIM III score (p < 0.05) from baseline to follow-up from median 68.0; IQR 10 (mean 68.7 ± 6.1; range 58–76) to 72.5; IQR 8 (mean 73.0 ± 6.1; range 59–88), with notable changes in scores in the mobility section (p < 0.05). Significant improvements in WISCI II and SCIM III scores were seen in participants with both motor complete and motor incomplete injuries, however the difference was highest in participants with incomplete SCI within the first 6 months (Table 3).

#### Outcomes of body function & structure

The International Standards for Neurological Classification of Spinal Cord Injury (ISNCSCI) lower extremity motor score (LEMS) changed significantly over the training programme (p < 0.05) with an average of 12% ± 3.8% improvement in LEMS reported for 7 of the 8 motor incomplete participants (Table 3). All other participants' scores were unchanged.

#### Participation outcomes

Overall psychosocial impact measured by the PIADS score at follow-up was positive (median 34, IQR 34; mean 36.1 ± 18.2), as well as in the three subscales, pertaining to “competence” (median 15, IQR 14; mean 15.1 ± 8.2), “adaptability” (median 10, IQR 7; mean 10.7 ± 4.8) and “self-esteem” (median 10, IQR 11, mean 10.4 ± 6.7). There was no significant difference between post-training and follow-up or between participants in the subacute vs. chronic stage. It can be noted that the overall PIADS score and all subscores were around the middle of the positive range (+ 1, + 2). Only a minor number of the subscores were reported negatively, ranging from 2.2% of the scores for “adaptability” to 4.0% of the scores for “competence” and 4.3% for “self-esteem”.

#### Outcomes of personal and environmental factors

**Fig. 8:**
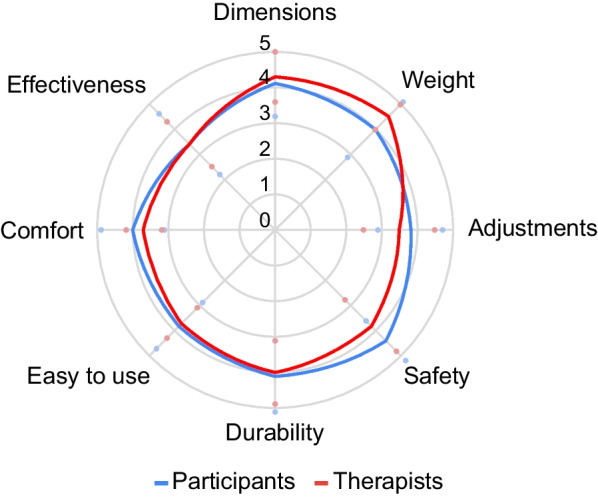
QUEST 2.0 mean scores for device satisfaction items. The figure shows the satisfaction with the device for participants and therapists at follow-up. Blue and red dots represent the standard deviation both for study participants’ and therapists’ scores, respectively

**Fig. 9:**
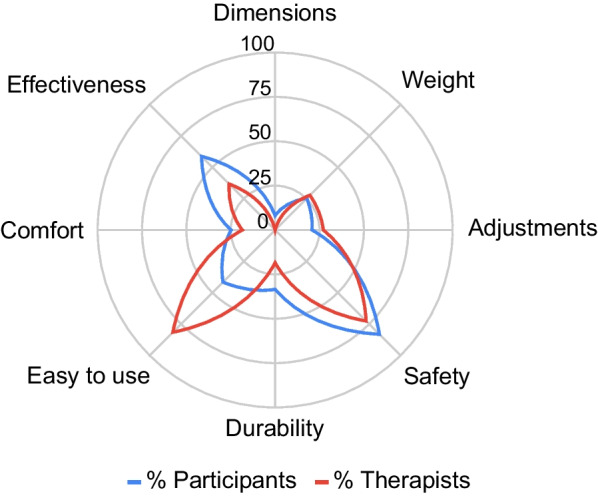
Importance of QUEST 2.0 categories for participants and therapists. The percentage of participants and therapists that selected each QUEST 2.0 category as the most important is shown

The median satisfaction with the exoskeleton was rated (total QUEST 2.0 score) as 33.0; IQR 7.5 (mean 31.7 ± 5.5) out of 40 at follow-up. No significant differences were found between visits when the QUEST 2.0 was performed (session 1, session 6, session 12 and follow-up). Results obtained for each of the rated items are shown below in Fig. 8. “Safety” was the best rated item (median 4.5, IQR 1.0; mean 4.4 ± 0.8), followed by “Durability” (median 4.0, IQR 1.0; mean 4.1 ± 1.0) and “Device dimensions” (median 4.0, IQR 1.0, mean 4.1 ± 0.9). “Safety” was also rated as the most important category for the majority of the participants (83.3%), followed by “Effectiveness” (58.3%) and “Ease of use” (41.7%) (see Fig. 9).

A total of 11 physiotherapists completed the QUEST 2.0, scoring an average total satisfaction with the exoskeleton of 31.6 ± 3.2 out of 40. The lightweight characteristic of the device (“Weight” category) appears to be the best rated category by therapists, followed by “Dimensions” and “Durability” (Fig. 8). “Ease of use” was rated as the most important category for the majority of therapists (83.3%), followed by “Safety” and “Effectiveness” (Fig. 9).

## Discussion

With the aim of achieving consistent comparability of our study results with other safety and feasibility studies of robotic lower-limb exoskeletons in persons with SCI, we based our protocol on the in- and exclusion criteria, training methodology and outcome measures most commonly used in previous exoskeleton trials (see Additional file 2 for an overview). We placed a special emphasis on the assessment of feedback from exoskeleton end-users as well as therapists.

The assessments used in our study were implemented with the aim of covering all domains of the ICF to ensure the possibility of analysing the meaningfulness of the results for individuals with SCI.

### Safety outcomes

A strict and exhaustive safety assessment was performed to overcome the reporting limitations of previous studies [9]. There are differences in the severity grading of AEs among studies, with events such as fractures not considered to be serious adverse events in every trial [10]. Thus, we took the most conservative approach and strictly reported all the adverse events that occurred during the study, including minor issues such as skin marking and minor bruising that may have been overlooked in previous studies. In this study, 8 device-related AEs were reported, with most of them (5/8) being of mild severity. Two device-related AEs were reported as a consequence of a device deficiency. In both of these cases, the participant sustained no lasting damage. Other device-related AEs in this study corresponded to skin lesions and pain. Skin lesions and pain in the areas of the body that are in direct contact with the device are well-known risks of the use of exoskeletons, with previous studies reporting between 2 and 13 device-related skin alterations when using similar devices with a comparable number of participants [1, 2, 16]. Episodes of orthostatic hypotension due to the standing up procedure also occurred in this study, however, these are to be expected given the propensity to this issue in the SCI population and have also been noted in other exoskeleton trials [4, 21]. As in previous studies, no falls occurred [1, 16]. These findings indicate that the ABLE Exoskeleton is comparable to other devices in regard to safety.

### Feasibility and usability outcomes

The mean average time to don and doff the ABLE Exoskeleton device was 6 min 50 s. This finding constitutes an improvement with respect to other exoskeletons that reported donning and doffing times averaging between 9 and 30 min, suggesting that the use of the ABLE Exoskeleton is feasible within the timeframes available in real-world rehabilitation settings [2, 6, 13, 24]. Moreover, as participants progressed through the training programme with the exoskeleton, the amount of assistance required to perform both the donning and doffing of the device decreased. The majority of participants were able to complete this process either independently, with minimum assistance, or with supervision by the end of the training programme. Similarly, a study investigating the effort to learn to use the Ekso™ exoskeleton found that 3/7 participants could independently don the device by the end of study [13]. Although a direct comparison of outcomes of exoskeleton studies is generally challenging due to the differences in the design of the devices (e.g. the ABLE Exoskeleton investigated in this study is only knee powered while the EksoGT^TM^ is hip-knee powered), we believe that it is valid to compare devices in respect to usability aspects.

By the end of training, 15 of 16 participants were able to complete all four activity tasks (sit-to-stand, walk 10 m, turn 180° and stand-to-sit) with the ABLE Exoskeleton, with 8 of them able to do so with minimum assistance, supervision or complete independence. Moreover, all participants showed a reduction in assistance needed to complete these tasks by the end of the training programme. In 77.7% of cases, training was delivered with the supervision of only one physiotherapist, showing the potential of the ABLE Exoskeleton to reduce the human resources required to deliver functional gait training in persons with complete SCI. However, the sit-to-stand transfer required most assistance compared to the other tasks. The reason for this is that the knee motors are not strong enough to complete the stand-up process without active arm and trunk support of the participant. Other studies with the Ekso™ device also reported a reduction in assistance needed as training progressed. Six out of 7 participants managed to walk in the Ekso™ device for 30 min with minimal assistance in a median of 8 sessions, with 5 of them able to do so with a contact guard or close supervision assistance in a median of 15 sessions [13]. Another study reported minimum assistance or less for walking with the Ekso™ device at the end of an 18-session training programme, whilst a need for moderate or maximum assistance was required for all sit-stand transitions [21]. As presented in our study, all participants increased their level of independence to complete all four therapy activities throughout the training programme, demonstrating how the ABLE Exoskeleton can be used as a tool during rehabilitation in clinical settings for locomotion training, with increasing independent use.

Participants’ walking time at the end of training was 2.6 times longer than at the start, indicating an increasing tolerance for gait training with the ABLE Exoskeleton, which is crucial to deliver intensive therapy as soon as possible after the injury in our cohort which contained mainly participants in the acute or subacute stage.

Results from the device metrics in this study were in keeping with other exoskeleton studies, with increases in the average number of steps (300%), speed (180%) and distance (290%) respectively from session 2 to 11. Similarly, studies with the Ekso™ device also reported an increase in the number of steps of 249% within 18 training sessions and 352% within a 25 session training programme, respectively [1, 21]. The wide range and standard deviation of the results in our study could be caused by our broad in- and exclusion criteria, which allowed us to recruit acute, subacute and chronic individuals with a wide range of motor impairments. However, as our results show, participants improved significantly over the course of the gait training programme in all gait parameters, similar to previous studies.

#### Outcomes of participation and personal/environmental factors

The average satisfaction score obtained for the ABLE Exoskeleton (31.6 ± 5.7) in study participants was comparable to the EksoGT™ exoskeleton (31.3 ± 5.70) [26] and the ReWalk device (29.4 ± 2.5) [32] when tested in individuals with multiple sclerosis, and considerably better than the Marsi Active Knee device (22.4 ± 3.2) when used by individuals with stroke and multiple sclerosis [27]. In respect to weight (Median: 4.0/Mean: 4.0 ± 1.0), easiness to use (Median: 4.0/Mean: 3.8 ± 0.9) and safety (Median: 4.5, Mean 4.4 ± 0.8) the ABLE Exoskeleton was also better in comparison to the Marsi Active Knee device (Weight: Median 2.8; Easiness to use: Median 2.6; Safety: Median 3.6) and comparable to the EksoGT™ device (Weight: Mean 3.8 ± 1.0; Easiness to use: Mean 3.7 ± 0.9; Safety: Mean 4.3 ± 0.9) [26, 27]. These results show that participants are satisfied with the ABLE Exoskeleton and its usability. The specific characteristics of the ABLE Exoskeleton of being lightweight and of smaller size were rated best by both participants and therapists. Participants rated their satisfaction highest in the QUEST 2.0 with respect to safety when using the device, while therapists scored it slightly lower. This finding indicates that participants are more satisfied with the safety of the device than the therapists. A reason for this could be that the therapists answered the QUEST after completing sessions with several participants where they had to provide different levels of assistance, in contrast to the participants who rated only their own experience. However, despite the difference in satisfaction scores between therapists and participants, the overall level of satisfaction scores amongst therapists still suggests a high acceptance of the ABLE Exoskeleton, which represents an essential prerequisite for its integration into clinical gait training programmes. To the best of our knowledge, this is the first study where the QUEST 2.0 was used to evaluate the satisfaction with the device among clinical professionals. Therapists represent an important stakeholder group responsible for the successful implementation of new technology into clinical practice [33].

The findings of the psychosocial impact assessment (PIADS) in this study suggest that the ABLE Exoskeleton, used in a clinical setting, may have a positive impact on quality of life and well-being, to be added to the more visible gait and motor function improvements. It should be noted that the PIADS is more relevant to persons in the chronic stage of SCI. In our study, no differences in the PIADS results between participants in the subacute and chronic phase after SCI were seen, but as there were only three participants in the chronic group, no conclusions can be made on this. Future studies should investigate the psychosocial impact of exoskeleton use on individuals with chronic SCI.

### Activity outcomes

Although demonstrating the efficacy of the ABLE Exoskeleton was not the primary endpoint of this study, significant improvements for outcomes on exertion and gait were measured by the BORG-scale, 10MWT, 6MWT and TUG performed with the exoskeleton were achieved and are consistent with the results from other devices [6]. Similarly, improved LEMS scores seen after training in participants in this study with motor incomplete lesions were also reported in previous exoskeleton trials [4, 22]. Besides the functional aspects, the average value of the BORG-scale indicates that the training with the ABLE Exoskeleton represents a moderate-intensity physical exercise with an expected positive effect on the cardiovascular system. However, another clinical study comparing the knee-powered ABLE Exoskeleton with Knee-Ankle-Foot orthoses (KAFOs) found no significant difference of performance in these walking tests. This study proposes that providing powered assistance only on the knee joints is not enough to provide sufficient trunk stability and reduced metabolic costs [11]. We also identified the lack of trunk stability in cervical and high thoracic lesions, but also the presence of spasticity in leg muscles and missing proprioception as main factors limiting the exoskeleton therapy outcome. The knowledge gained in this clinical study in this regard led to some changes in the design of the ABLE Exoskeleton, which now includes motor-powered hip joints, optional shoulder straps and an articulated ankle joint, to increase the support provided to the trunk and therefore extend the group of potential end users.

Furthermore, the significant improvement in the WISCI II scores seen in this study shows that participants also developed less dependency on walking aids without the exoskeleton [29]. Another study also reported significant changes in walking ability within an 8-week training programme with the Ekso™ device in recently injured participants [4]. Positive changes in the SCIM III were seen after training in this study and have also been identified post-training in a 2-week training programme by a study with the AIDER exoskeleton, indicating improvements in the degree of independence to complete activities of daily living [22]. Whilst it is not possible to conclude that all of these changes have occurred only as a direct result of exoskeleton training due to a number of confounding variables and lack of a control group, they do suggest that the participants became more tolerant to gait training in the device, were able to master the basic technique of walking more efficiently by the end of training, and had an improved function outside of training sessions. These results suggest that gait training with the ABLE Exoskeleton has the potential to become an effective tool for improving standing and walking function of individuals with SCI in combination with conventional rehabilitation techniques.

### Study limitations

There were several limitations to this study. Firstly, this was an open and non-blinded/non-controlled study, therefore selection bias towards physically fit and cooperative participants was present. This limits the generalisation of the results to the SCI population as a whole. The heterogeneity of the study population together with the relatively small sample size lowered the statistical power in calculations. Furthermore, the primary endpoint of this study was on safety and feasibility and not on efficacy. Since many study participants were in the acute and subacute phase after injury, during which spontaneous recovery occurs, no causal relationship between the observed improvements and the exoskeleton use can be made. Finally, due to the COVID-19 pandemic, there was reduced access to potential participants who could have participated, with both centres initially unable to recruit outpatients and also a reduction of in-patients being admitted for neurorehabilitation. Despite the challenging circumstances we were still able to recruit 24 participants with a wide range of motor impairments and time after SCI.

## Conclusions

In conclusion, the results of our multicentre study show that the ABLE Exoskeleton is safe and feasible for gait training in persons with SCI with neurological injury levels ranging from C5 to L3 in a rehabilitation hospital setting, regardless of the time since injury (acute or chronic) and its severity (motor complete or incomplete SCI). Similarly, it was feasible for all the participants to don and doff the device within an efficient timeframe, which maximises the effective gait training time of each rehabilitation session. The degree of independence to complete therapy activities improved throughout the training, whilst also making progress with gait performance. Furthermore, the protocol formulated for this study has intended to bring together the key aspects of previous safety and feasibility trials with lower-limb exoskeletons. Thus, we were able to maximise the comparability of our results with these previous studies. It is hoped that our study protocol can serve as a framework for future exoskeleton trials to allow for more consistency and comparability of results. Such a framework is not only beneficial for the early evaluation of a specific exoskeleton against other types of exoskeletons and/or standard rehabilitation interventions for persons with SCI, but also for the assessment of its impact on function and health in a community-based setting outside the hospital.

## Supplementary Information

**Additional file 1.** Literature review results: This document provides the detailed results of a literature review of studies that evaluated the safety, feasibility, and usability of exoskeletons for the SCI population, which was conducted on PubMed, Science Direct, Cochrane library, DOAJ and BMJ between the 7th and 30th January 2020 for studies completed since 1 January 2010.

**Additional file 2.** Recommendations for clinical study protocols of early studies with robotic lower limbs exoskeletons. This document lists the recommended inclusion/exclusion criteria, training methodology and outcome measures as a framework for future clinical trials to assess the safety, feasibility and usability of robotic lower-limb exoskeletons in individuals with SCI.

## Data Availability

The datasets used and analysed during the current study are available from the corresponding author on reasonable request.

## Abbreviations

10MWT: 10 Metre Walk Test
6MWT: 6 Minute Walk Test
AD: Autonomic dysreflexia
AE: Adverse events
AIS: American Spinal Injury Association Impairment Scale
CVS: Cardiovascular system
DBP: Diastolic blood pressure
DVT: Deep venous thrombosis
DXA: Dual Energy X-Ray Absorptiometry
EPUAP: European Pressure Ulcer Advisory Panel
ICF: International Classification of Functioning, Disability and Health
ISNCSCI: International Standards for Neurological Classification of Spinal Cord Injury
IQR: Interquartile range
LEMS: Lower Extremity Motor Score
LoA: Level of Assistance
PIADS: Psychosocial Impact of Assistive Devices Scale
PROMs: Patient-Reported Outcome Measures
QoL: Quality of life
QUEST: Quebec User Evaluation of Satisfaction with assistive Technology
ROM: Range of Motion
RPE: Rate of perceived exertion
SAE: Serious adverse events
SBP: Systolic blood pressure
SCI: Spinal cord injury
SCIM: Spinal cord independence measure
STROBE: Strengthening the Reporting of Observational Studies in Epidemiology
SD: Standard deviation
TUG: Timed up and go
VAS: Visual Analogue Scale
WISCI: Walking Index for Spinal Cord Injury
