# Supplementary material for: Multicentric investigation on the safety, feasibility and usability of the ABLE lower-limb robotic exoskeleton for individuals with spinal cord injury: a framework towards the standardisation of clinical evaluations
Source: J Neuroeng Rehabil. 2023 Apr 12;20:45. doi: 10.1186/s12984-023-01165-0 (PMC10091314; doi:10.1186/s12984-023-01165-0)
Supplement: Supplementary file 1 — Additional file 1. Literature review results: This document provides the detailed results of a literature review of studies that evaluated the safety, feasibility, and usability of exoskeletons for the SCI population, which was conducted on PubMed, Science Direct, Cochrane library, DOAJ and BMJ between the 7th and 30th January 2020 for studies completed since 1 January 2010. [file 12984_2023_1165_MOESM1_ESM.docx]

**ADDITIONAL MATERIAL I - LITERATURE REVIEW**

A literature review of studies that evaluated the safety, feasibility, and usability of exoskeletons for the SCI population was conducted between the 7^th^ and 30^th^ January 2020 for studies completed since 1 January 2010. Searches were conducted on PubMed, Science Direct, Cochrane library, DOAJ and BMJ. The following search terms were used in all fields: "Exoskeleton" AND "SCI" AND "Gait" AND ("Feasibility" OR "Safety" OR “Usability”). A manual search on Google scholar for known exoskeletons and a search of study reference lists was also conducted. A total of 204 records were identified. After removing duplicate references, 103 studies were assessed by their title and secondly by their abstracts, leading to 82 records being excluded. After screening, 21 studies were full-text assessed for eligibility and a total of 13 studies were finally included in the review.

**Table S1.** List of studies included in the literature review

| 1. | McIntosh K, Charbonneau R, Bensaada Y, Bhatiya U, Ho C. The Safety and Feasibility of Exoskeletal-Assisted Walking in Acute Rehabilitation After Spinal Cord Injury. Archives of Physical Medicine and Rehabilitation. 2020;101(1):113-20. |
| --- | --- |
| 2. | Tefertiller C, Hays K, Jones J, Jayaraman A, Hartigan C, Bushnik T, et al. Initial Outcomes from a Multicenter Study Utilizing the Indego Powered Exoskeleton in Spinal Cord Injury. Topics in Spinal Cord Injury Rehabilitation. 2018;24(1):78-85. |
| 4. | Bach Baunsgaard C, Vig Nissen U, Katrin Brust A, Frotzler A, Ribeill C, Kalke YB, et al. Gait training after spinal cord injury: safety, feasibility and gait function following 8 weeks of training with the exoskeletons from Ekso Bionics. Spinal Cord. 2018;56(2):106-16. |
| 6. | Kolakowsky-Hayner S. Safety and Feasibility of using the EksoTM Bionic Exoskeleton to Aid Ambulation after Spinal Cord Injury. Journal of Spine. 2013;4. |
| 12. | Kozlowski AJ, Bryce TN, Dijkers MP. Time and Effort Required by Persons with Spinal Cord Injury to Learn to Use a Powered Exoskeleton for Assisted Walking. Topics in Spinal Cord Injury Rehabilitation. 2015;21(2):110-21. |
| 13. | Wu CH, Mao HF, Hu JS, Wang TY, Tsai YJ, Hsu WL. The effects of gait training using powered lower limb exoskeleton robot on individuals with complete spinal cord injury. Journal of Neuroengineering and Rehabilitation. 2018;15(1):14. |
| 14. | Spungen AM, Asselin PK, Fineberg DB, Kornfeld SD, Harel NY, editors. Exoskeletal-Assisted Walking for Persons with Motor-Complete Paraplegia. North Atlantic Treaty Organization (NATO), Research and Technology Organization, Human Factors and Medicine Panel, HFM-228 Symposium; 2013 April 15-17; Milan, Italy. |
| 15. | Yang A, Asselin P, Knezevic S, Kornfeld S, Spungen AM. Assessment of In-Hospital Walking Velocity and Level of Assistance in a Powered Exoskeleton in Persons with Spinal Cord Injury. Topics in Spinal Cord Injury Rehabilitation. 2015;21(2):100-9. |
| 16. | Sale P, Russo EF, Russo M, Masiero S, Piccione F, Calabro RS, et al. Effects on mobility training and de-adaptations in subjects with Spinal Cord Injury due to a Wearable Robot: a preliminary report. BMC Neurology. 2016;16:12. |
| 19. | Gagnon DH, Escalona MJ, Vermette M, Carvalho LP, Karelis AD, Duclos C, et al. Locomotor training using an overground robotic exoskeleton in long-term manual wheelchair users with a chronic spinal cord injury living in the community: Lessons learned from a feasibility study in terms of recruitment, attendance, learnability, performance and safety. Journal of Neuroengineering and Rehabilitation. 2018;15(1):12. |
| 20. | Xiang XN, Ding MF, Zong HY, Liu Y, Cheng H, He CQ, et al. The safety and feasibility of a new rehabilitation robotic exoskeleton for assisting individuals with lower extremity motor complete lesions following spinal cord injury (SCI): an observational study. Spinal Cord. 2020;58(7):787-794. |
| 21. | Hartigan C, Kandilakis C, Dalley S, Clausen M, Wilson E, Morrison S, et al. Mobility Outcomes Following Five Training Sessions with a Powered Exoskeleton. Topics in Spinal Cord Injury Rehabilitation. 2015;21(2):93-9. |
| 22. | Birch N, Graham J, Priestley T, Heywood C, Sakel M, Gall A, et al. Results of the first interim analysis of the RAPPER II trial in patients with spinal cord injury: ambulation and functional exercise programs in the REX powered walking aid. Journal of Neuroengineering and Rehabilitation. 2017;14(1):60. |
