# Supplementary material for: Multicentric investigation on the safety, feasibility and usability of the ABLE lower-limb robotic exoskeleton for individuals with spinal cord injury: a framework towards the standardisation of clinical evaluations
Source: J Neuroeng Rehabil. 2023 Apr 12;20:45. doi: 10.1186/s12984-023-01165-0 (PMC10091314; doi:10.1186/s12984-023-01165-0)
Supplement: Supplementary file 2 — Additional file 2. Recommendations for clinical study protocols of early studies with robotic lower limbs exoskeletons. This document lists the recommended inclusion/exclusion criteria, training methodology and outcome measures as a framework for future clinical trials to assess the safety, feasibility and usability of robotic lower-limb exoskeletons in individuals with SCI. [file 12984_2023_1165_MOESM2_ESM.docx]

# **ADDITIONAL MATERIAL II – RECOMMENDATIONS FOR CLINICAL STUDY PROTOCOLS OF EARLY STUDIES WITH ROBOTIC LOWER LIMB EXOSKELETONS**

| **Framework for clinical trials to assess the safety, feasibility and usability of robotic lower-limb exoskeletons in individuals with SCI** | | | |
| --- | --- | --- | --- |
| **Inclusion criteria** | | **Exclusion criteria** | |
| - 18 to 70 years of age - Traumatic and non-traumatic SCI - Inpatient or outpatient - American Spinal Injury Association Impairment Scale (AIS) A to AIS D with sufficient arm strength to support body weight on a walking frame | | - WISCI II without exoskeleton of >16 - Body measurements incompatible with the device - 5 or more risk factors for fragility as stated by Craven et al., Topics in Spinal Cord Injury Rehabilitation 14:1-22, 2009 - Severe comorbidities / health-related aspects that are not considered to be appropriate to complete study participation - Unable to perform a sit-to-stand transfer or stand in the device with assistance | |
| **Training methodology** | | | |
| - Total session number: 12 - Frequency: 3x/week for 4-6 weeks - Session duration: 60 minutes, incl. adjustments, donning/ doffing and data collection time - Therapy time: > 30 minutes of time spent sitting, standing or walking with the exoskeleton - Content of the training programme:   o First sessions: basic techniques to use the exoskeleton  o Every session: predefined activity tasks: (1) sit-to-stand, (2) walk 10 metres, (3) turn 180° and (4) stand-to-sit | | | |
| **Outcome measures** | | | |
| ***Safety*** | ***Feasibility*** | | ***Usability*** |
| - Number of device-related (Severe) Adverse Events - Number of drop-outs due to the device - Number of falls | - Time to don/doff the device - Level of Assistance (LoA) to don/doff the device - LoA to complete activity tasks | | - Number of steps, speed and distance walked with the exoskeleton - Walking time and standing time with the exoskeleton |
| **Secondary Outcomes** | | | |
| ***Body function & structure*** | ***Activities*** | | ***Participation, personal & environmental factors*** |
| - ISNCSCI | - Walking Tests: TUG, 10MWT, 6MWT, WISCI II - BORG - SCIM III | | - PIADS - QUEST 2.0 with participants and therapists |

Abbreviations defined in List of Abbreviations section of the main paper.
